# Self-other control: a candidate mechanism for social cognitive function

**DOI:** 10.3389/fnhum.2014.00789

**Published:** 2014-10-08

**Authors:** Sophie Sowden, Punit Shah

**Affiliations:** MRC Social, Genetic and Developmental Psychiatry Centre, Institute of Psychiatry, Psychology and NeuroscienceKing's College London, UK

**Keywords:** self-other control, social cognition, autism, schizophrenia, right temporoparietal junction, neuropsychological markers

Despite ever-growing interest in the “social brain” and the search for the neural underpinnings of social cognition, we are yet to fully understand the basic neurocognitive mechanisms underlying complex social behaviors. One such candidate mechanism is the control of neural representations of the self and of other people (Brass et al., 2009; Spengler et al., 2009a), and it is likely that “common” disorders of social cognition such as autism and schizophrenia involve atypical modulation of self and other representations (Cook and Bird, 2012; Ferri et al., 2012). This opinion piece will first consider self-other control as a possible low-level neurocognitive mechanism for social functioning across many domains of social cognition. Neuroscientific evidence will be drawn upon and the potential for a better understanding and identification of neuropsychological markers for atypical social cognitive development, discussed.

## A candidate mechanism

Humans are uniquely social beings and therefore identifying commonalities in the mechanisms recruited across various domains of social cognition is important, providing an understanding not only of typical social cognitive function but also what happens when this goes wrong. A candidate process which may be recruited across a range of socio-cognitive tasks is the ability to hold in mind and manage neural representations of both the self and of other people. Motor representations pertaining to the self and of the other are necessary in the case of imitation (di Pellegrino et al., 1992; Gallese et al., 1996), and self and other representations of mental and affective states are necessary for theory of mind and empathy, respectively (Decety and Grèzes, 2006; Brass and Spengler, 2008; Iacoboni, 2009). Within each of these domains of social cognition a form of “contagion” can be observed where information is shared between representations of the self and other. In the case of action observation, individuals automatically and often non-consciously imitate the actions of those with whom they interact (Chartrand and Bargh, 1999; Brass et al., 2000; Heyes, 2011).

Social interaction therefore appears to be facilitated by a shared representational system. However, social situations sometimes require an individual to distance themselves from other people and in other instances require one to engage more with representations of others. For example, when taking another's perspective, engaging a successful theory of mind, or empathizing with others it is important to put aside or inhibit one's own perspective, mental or affective state and enhance that of the interacting other. Conversely, in order to control the tendency to imitate others' actions and generate our own independent actions, we must inhibit the motor representation pertaining to the interacting other and activate the motor representation for our own intended action. Differing requirements to inhibit or enhance representation of the self or the other for successful social interaction highlights the crucial role played by the ability to *control* or *switch between* neural representations attributed to the self and to other people, hereafter referred to as “self-other control” (Decety and Sommerville, 2003; Brass and Heyes, 2005; Spengler et al., 2009a).

**Figure 1:**
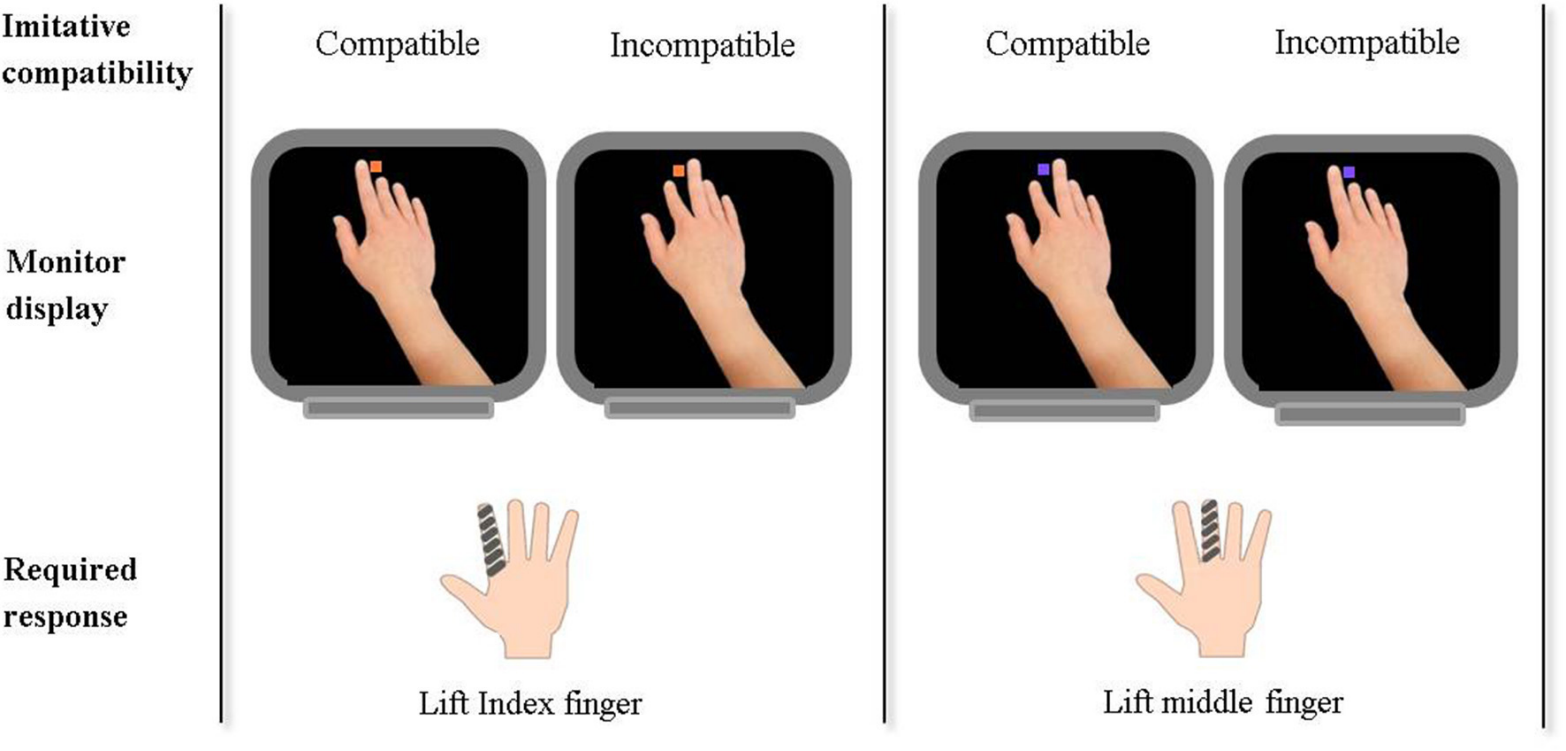
**Example of stimuli presented and participant responses required in a task to measure the control of imitation**. The task: Participants are instructed to make either an index or middle finger lifting action in response to a colored cue (orange or purple square) presented on the monitor display. The cue is also accompanied by a task-irrelevant hand performing an index or middle finger lifting action. Thus, task-irrelevant stimuli can be either imitatively compatible or incompatible with the required finger response. An index of self-other control is calculated by subtracting response times on compatible trials from those on incompatible trials. Imitative compatibility of the task-irrelevant stimuli with the required finger lift response is also indicated for trials in which an orange square indicates lift index and a purple square indicates lift purple.

A task now readily used as a behavioral index of self-other control is that of the control of imitation (Figure 1; Brass et al., 2001, 2005, 2009; Spengler et al., 2009a; Catmur and Heyes, 2011; Santiesteban et al., 2012a,b; Sowden and Catmur, 2013). The task requires participants to inhibit imitative response tendencies, and therefore provides an index of an individual's ability to enhance the self-representation whilst inhibiting the other-representation. Additionally, Obhi and Hogeveen (2013) have proposed a complimentary task whereby performance under the opposite control requirements can be investigated; *inhibiting* the self-representation whilst exciting the other-representation. In combination, these tasks provide a neat index of control, the ability to supress not only representations of the other but also of the self.

Despite the very different higher-level cognitive processes involved in a wide range of social cognitive abilities, a series of behavioral findings in neurotypical adults support the existence of a common low-level mechanism of self-other control. Performance in one social domain such as the control of imitation correlates highly with performance in other social domains requiring self-other control. These include perspective-taking, theory of mind and empathy (Spengler et al., 2010a), and remain even when controlling for more general executive functioning processes (e.g., Spengler et al., 2010b). The link between performance on different tasks requiring self-other control is not merely correlational; training to inhibit imitation produces an enhancement of perspective-taking abilities (Santiesteban et al., 2012b). Moreover, priming pro-social attitudes enhances automatic imitation but not a non-imitative control process (Leighton et al., 2010; Cook and Bird, 2011) and engaging in more social interaction appears to improve other social abilities (Hogeveen and Obhi, 2012). Both of these examples support the enhancement of a common process involved in social functioning.

## A neural basis for self-other control

As well as the medial prefrontal cortex (mPFC), the right temporoparietal junction (rTPJ), a brain region located at the intersection of the superior temporal sulcus and inferior parietal lobule, has attracted extensive research attention and has now been implicated in a wide range of social cognitive abilities, including judging agency, perspective-taking, theory of mind and empathy (Decety and Sommerville, 2003; Decety and Lamm, 2007; van Overwalle, 2009; Sperduti et al., 2011). A series of studies by Brass et al. (2001, 2005, 2009) and Spengler et al. (2009a,b) utilized functional magnetic resonance imaging (fMRI) to localize the neural areas related to the control of imitation to the rTPJ and mPFC. These studies suggest that the mPFC and/or the TPJ may be the neural substrate of self-other control.

Further, causal evidence for the role of the rTPJ in self-other control is derived from studies measuring the effects of magnetic or electric stimulation of this area. Disruptive repetitive transcranial magnetic stimulation (rTMS) of rTPJ has been shown to impair performance in both the control of imitation (Sowden and Catmur, 2013) and theory of mind (Costa et al., 2008; Young et al., 2010). Excitatory transcranial direct current stimulation (tDCS) enhanced imitative control and perspective-taking performance (Santiesteban et al., 2012a). The work of Santiesteban and colleagues is particularly noteworthy, as excitation of rTPJ enhanced self representations and inhibited representation of the other in the imitation inhibition task, but also enhanced other representations and inhibited self representations in the perspective-taking task. This pattern of results is best explained by the up-regulation of a mechanism which facilitates the *control* of self and other representations. Similarly, acquired temporoparietal lesions have been associated with rare disorders such as asomatognosia, characterized by the misidentification of part of one's own body as belonging to another (Feinberg et al., 2010) and anosognosia, characterized by a denial or unawareness of a paralyzed limb (Ramachandran and Blakeslee, 1998).

Another competing idea is that the mPFC and rTPJ, rather than facilitating the control of competing representations of self and other, may in fact help to differentiate task-relevant from task-irrelevant representations (Nicolle et al., 2012; Cook, 2014). Indeed, there may be an interesting avenue for picking apart these two dimensions. However, at present it remains unclear how this mechanism may extend to a range of social cognitive abilities investigated to date in the self-other control literature, and how this may translate into a mechanism capable of explaining atypical social cognition.

## Atypical social cognitive development

Uncovering a common low-level mechanism for social cognition seems particularly useful when considering atypical social cognitive development. Mirror touch synaesthesia, in which the observation of touch or pain to others elicits an overt somatic sensation in the synaesthete's own body, is also associated with structural abnormalities in the TPJ and could be described as one example of a disorder of self-other control (Banissy and Ward, 2013; Holle et al., 2013).

Similarly, the ability to control neural representations of the self and of other people seems a central aspect of more common disorders of social cognition, such as autism and schizophrenia (Spengler et al., 2010b; Ferri et al., 2012). Several studies postulate atypical self-control in these disorders which impacts upon the attribution of agency to self and others in individuals with schizophrenia (Renes et al., 2013), and impairments in inhibiting imitation, theory of mind and perspective-taking in ASD (Lombardo et al., 2010, 2011; Spengler et al., 2010a,b). Lombardo et al. (2011) identified abnormalities in the recruitment of the rTPJ when making judgments requiring self-other differentiation in individuals with ASD relative to controls. Similarly, Spengler et al. (2010b) found that, in a sample of high functioning autistic individuals, increased imitation was associated with reduced theory of mind and decreased activity in areas typically required for self-other control. Despite varied terminology, including self-other “differentiation,” “distinction,” “switching” or “agency,” all postulated processes appear to share a common feature of the “control” of shared representations.

Indeed, key aspects of the schizophrenia symptom profile can be explained by a deficit in self-other control. Identity and reality disturbances including hallucinations and delusions of persecutory control, disturbed consciousness and thought insertion exemplify a misattribution of self-generated, internal representations to others or the external world, highlighting a difficulty in managing representations of self and others (Frith, 1992; Allen et al., 2004, 2006; Jeannerod, 2009). Moreover, abnormal structure and function of the TPJ is reported in individuals at risk (Brüne et al., 2011), as well as suffering from schizophrenia (Benedetti et al., 2009; Lee et al., 2011; Das et al., 2012; de Achával et al., 2012; Koeda et al., 2013), relative to healthy controls. Diminished activation of this region has been associated with impaired social cognitive performance, in particular theory of mind and emotion processing domains (Benedetti et al., 2009; Lee et al., 2011; Das et al., 2012).

More recently it has been suggested that the impairments seen in ASD and schizophrenia can be characterized as a failure of top-down modulation of social behavior (Southgate and Hamilton, 2008; Cook and Bird, 2012; Cook et al., 2012; Wang and Hamilton, 2012). Of particular note, Cook and Bird (2012) found that the modulatory effects of priming pro-social attitudes on self-other control observed in neurotypical adults were absent in individuals with ASD. In the same vein, reduced fronto-temporal functional connectivity is now a well-established feature of schizophrenia and has been linked to diminished top-down modulatory control over social behavior (Allen et al., 2008; Cook et al., 2012).

## A neurocognitive marker for atypical social cognition?

Although we know little about the precise developmental trajectories for the neurocognitive deficits discussed, by highlighting a mechanism with the potential to explain many facets of social cognitive function researchers may be better equipped to advise on neurocognitive markers and possible interventions for common disorders of social cognition. Self-other control emerges as such a candidate neurocognitive mechanism. Future assessment of disorders of social cognition can benefit from the now widely used task of imitative control (Figure 1) as a robust behavioral index of self-other control which includes the requirement for online modulation of both self- and other-representations in one task. Performance on imitative control tasks predicts performance across various domains of social cognition, and thus may provide a means to predict a pattern of atypical social development, in addition to measures of the structure and function of critical regions such as the rTPJ and mPFC. One may predict that individuals with autism or schizophrenia will be impaired at controlling imitative response tendencies, indicative of a deficit in self-other control.

This opinion piece has explored behavioral and neuroscientific evidence for self-other control as a candidate neurocognitive mechanism for social cognition. With advances in the field, a mechanism such as this may be crucial in identifying neurocognitive markers of atypical development and providing a therapeutic target to ameliorate the symptoms of atypical social development. Of particular promise from the application of such a mechanism is a unified account of the broad range of social functioning impairments associated with ASD and schizophrenia.

### Conflict of interest statement

The authors declare that the research was conducted in the absence of any commercial or financial relationships that could be construed as a potential conflict of interest.
